# Environmental Risks of Antibiotics and Antibiotic Resistance Elements: Occurrence, Fate, and Assessment

**DOI:** 10.3390/ijms27073255

**Published:** 2026-04-03

**Authors:** Fiaz Ahmad, Azzam Fatima Zahra, Noreen Ashraf, Zafar Iqbal

**Affiliations:** 1 Key Laboratory for Space Bioscience & Biotechnology, School of Life Science and Technology, Northwestern Polytechnical University, Xi’an 710129, China; 2 Central Laboratories, King Faisal University, Al-Ahsa 31982, Saudi Arabia

**Keywords:** antibiotic resistance, environmental resistome, risk assessment, public health, dissemination

## Abstract

Antibiotics, antibiotic-resistant bacteria (ARB), and antibiotic resistance genes (ARGs) have emerged as critical environmental contaminants posing serious ecological and public health concerns. The widespread occurrence and proliferation of ARB and ARGs in wastewater treatment plants (WWTPs) and reclaimed wastewater (RWW) used for irrigation represent major pathways for their dissemination into the environment. Current knowledge indicates that ARGs from the environmental resistome can be transferred among diverse microbial communities, including clinically relevant human pathogens. Numerous studies have also linked the expansion of the environmental resistome to anthropogenic activities. Therefore, preventing and mitigating the spread of antibiotic resistance in the environment requires a deeper understanding of how resistance genes evolve, transfer, and persist across ecological compartments. This review synthesizes the current state of knowledge on the occurrence, prevalence, and detection of antibiotics, ARB, and ARGs in various environmental matrices, providing essential insights for developing preventive strategies and promoting the sustainable management of ecosystems.

## 1. Introduction

Antibiotics, whether produced synthetically or existing naturally, play a pivotal role in modern medicine, agriculture, and veterinary practices due to their capacity to prevent and treat infections in humans, plants, and animals. Since their first widespread use in the 1940s, antibiotics have become deeply integrated in health care and agricultural systems [1,2,3,4]. Over the past decades, global demand for antimicrobials has escalated dramatically. This increase is driven by population growth, intensified livestock production, and expanded clinical usage, resulting in production volumes reaching thousands of tons per year [5,6,7,8].

This surge in antibiotic use has coincided with growing evidence of the release and environmental dissemination of ARB and ARGs, which are collectively referred to as antibiotic resistance elements. These elements have been detected in WWTPs, RWW used for irrigation, soils, and sediments, and within biota [9,10,11,12]. Indeed, the anthropogenic origin and spread of ARGs have been linked to diverse human activities, for example, pharmaceutical manufacturing, the improper disposal of unused medications, the use of antimicrobials in animal husbandry, and the discharge of hospital effluents [13,14]

A significant fraction of administered antibiotics escapes metabolism of the host. However, this escape differs depending on the class and host metabolism; 30% to 90% of antibiotic doses may be excreted (unaltered or as active metabolites) in urine and feces [11,15,16,17,18,19]. These unmetabolized residues thus serve as input to sewage systems, and in agricultural settings, antibiotics and ARGs, harboring microbes may further enter the environment through excretion from grazing livestock or via application of untreated manure to soil [20].

The reuse of RWW for irrigation is increasingly viewed as a significant conduit for the transmission of ARB and ARGs into agricultural soils and crops [21,22]. Conventional wastewater treatment processes, such as activated sludge, membrane bioreactors, and moving-bed biofilm reactors, often fail to fully eliminate antibiotics, ARB, or ARGs; therefore, residual loads persist into the effluent [9,23,24,25]. To reduce these residuals further, advanced or tertiary treatments are sometimes employed, for example, activated carbon adsorption, reverse osmosis, ozonation, Fenton oxidation, and ultraviolet disinfection [23,24,26,27]. However, these methods are more expensive and energy-intensive.

Once antibiotic residues and resistance elements reach soils, sediments, or plant rhizospheres, many studies have documented their ability to enter crops, as well as uptake by plants, or transport via soil pore water. Such transfer pathways raise concerns about entry into the food chain and ecological exposure [28,29,30]. Because RWW often carries both antibiotic compounds and high loads of ARB/ARGs, it constitutes an intensified burden to the receiving environment [31,32,33,34,35].

Wastewater treatment plants are especially critical zones for enhanced horizontal gene transfer (HGT). Such enhancement can be due to high microbial densities, disparate bacterial taxa, and selective pressures, effectively turning them into “genetic reactors” for ARG exchange [31]. Repeated use or application of RWW, manure, and biosolids in agricultural systems further fosters the expansion, persistence, and amplification of ARB and ARG populations in soils and crops [36,37,38]. The persistence of antibiotic resistance in ecosystems carries serious implications not only for human health but also for economic burdens and environmental resilience [39,40,41,42].

In addition, climate change and increasing water scarcity are exerting indirect pressures on how and where reclaimed water is used. Prolonged dry periods and dwindling freshwater supplies push agricultural stakeholders to rely more heavily on RWW, potentially exacerbating the spread of resistance elements [43]. Emerging research also suggests that rising temperatures, altered precipitation patterns, and other climate stressors may influence bacterial growth dynamics, selection pressure, and the mobilization of ARGs across environments [44].

Recent advances in detection techniques, especially metagenomics, high-throughput sequencing, and multi-omics approaches, have greatly expanded our ability to detect ARGs across entire microbial communities. This enables finer resolution of resistome structures and dynamics of microbial growth and the dissemination of resistance elements [45]. Moreover, large-scale databases on environmental ARG abundance are emerging, helping to benchmark global trends and assess the relative risk across ecosystems [46].

Given these developments, this review aims to synthesize the current state of knowledge regarding the prevalence, fate, and detection of antibiotics, ARB, and ARGs in environmental compartments. We examine risk assessment frameworks and pathways of resistance transmission, and we also highlight future research directions toward mitigation and sustainable management of antibiotic resistance in the environment.

## 2. Fate of ARB and ARGs in the Environment

### 2.1. Fate of ARB and ARGs in WWTPs

Numerous studies have documented the widespread presence of ARB and ARGs in WWTPs and their downstream environments, including RWW used for irrigation, and even in drinking water sources [47,48,49]. A well-documented example of the environmental and public health impact of antibiotic-resistant pathogens occurred in Europe during the summer of 2011, when an outbreak of multidrug-resistant *Escherichia coli* O104:H4 (enterohemorrhagic *E. coli*, EHEC) caused hemolytic–uremic syndrome (HUS) in Germany and France. This outbreak, traced to contaminated water-sprayed vegetables, resulted in 877 HUS cases (32 deaths) and 3043 EHEC infections (16 deaths) [50]. Recent years have seen a notable rise in ARB and ARG prevalence in WWTPs, partly attributed to the increased use of antibiotics during and after the COVID-19 pandemic. These situations intensified selective pressure on environmental microbiomes [51].

Recent studies increasingly highlight that aquatic environments directly linked to human exposure, particularly recreational waters, serve as important reservoirs and transmission pathways for ARB and ARGs. These environments are frequently impacted by wastewater inputs, facilitating the dissemination of fecal indicator bacteria such as *E. coli* and *Enterococcus*, which are widely recognized as key indicators of sanitary contamination and public health risk. Evidence shows that fecal indicator bacteria in surface waters not only persist but also exhibit diverse and spatially variable resistance profiles that are influenced by environmental and anthropogenic factors [52]. Moreover, recreational waters represent critical interfaces where human populations are directly exposed to ARB, including clinically relevant strains carrying mobile resistance elements, underscoring their role in environmental-to-human transmission pathways. Additional studies further demonstrate that aquatic systems contaminated with fecal and chemical pollutants can promote the development and spread of resistant microbiota, thereby amplifying risks to human health [53,54,55]. Collectively, these findings emphasize the need to explicitly incorporate fecal indicator bacteria and recreational water exposure pathways into assessments of antibiotic resistance in aquatic environments.

**Figure 1:**
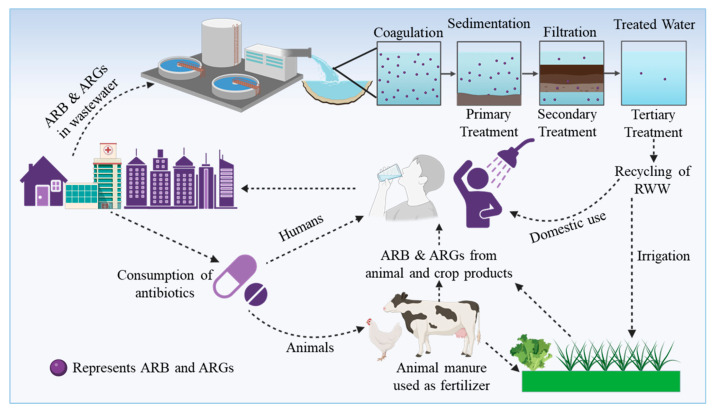
Schematic representation of the transmission pathways of antibiotic-resistant bacteria (ARB) and antibiotic resistance genes (ARGs) in the environment. Antibiotics administered to humans (through prescribed or unprescribed treatments) and animals (livestock and pets) are not fully metabolized, with up to 90% excreted via feces into wastewater. These residues contribute to the proliferation of ARB and ARGs in water systems, which may persist even after wastewater treatment (upper right). Contaminated water is subsequently reused for drinking, household activities, or agricultural irrigation, facilitating further transmission through crops, food products from treated animals, and direct contact with pets. This cycle perpetuates the environmental dissemination of resistance, posing serious risks to human health. Created in BioRender. Khalid, A. (2026) https://BioRender.com/anp7zv3.

WWTPs generally operate through three major treatment stages: (i) primary treatment, where solids are physically removed; (ii) secondary treatment, involving biological and chemical degradation of organic matter; and (iii) tertiary treatment, incorporating advanced physical–chemical processes for polishing and disinfection (Figure 1). Each phase significantly alters the bacterial community composition and abundance of resistance determinants [56]. However, even after conventional tertiary treatment, substantial loads of ARB and ARGs are frequently detected in effluents, sludge, and biofilms [57,58,59,60,61]. Proia et al. (2018) compared hospital and urban wastewaters and observed that, while the total abundance of ARGs decreased from influent to effluent, the relative abundance of ARB increased downstream of WWTP outfalls—these findings suggest selective survival or proliferation under treatment stress [62]. Similarly, Auerbach et al. (2007) found that WWTPs serve as hotspots for tetracycline-resistant genes, which persisted across all treatment compartments, with minimal reduction after processing [63]. Goñi-Urriza et al. (2000) demonstrated that discharge from WWTPs substantially increased resistance among *Enterobacteriaceae* and *Aeromonas* spp. in Spain’s Agra River, where isolates exhibited resistance to 21 out of 22 tested antibiotics [59]. In Beijing, Xu et al. (2016) reported elevated concentrations of sulfonamide and tetracycline residues, ranging from 1.3 × 10^1^ to 1.5 × 10^3^ ng L^−1^ and 3.9 × 10^1^ to 5.4 × 10^4^ ng L^−1^ in surface waters, respectively, and a higher abundance of ARGs detected in sediments [64]. Sulfonamide resistance was found to be 2–3 times more prevalent than tetracycline resistance, indicating differential persistence and selection among antibiotic classes (Table S1).

The design, operational parameters, and environmental conditions of WWTPs significantly affect the fate and persistence of ARB and ARGs [65,66,67]. For instance, Kim et al. (2007) reported that increasing organic load in biological treatment units enhanced the growth of tetracycline-resistant bacteria, emphasizing that nutrient levels and hydraulic retention time modulate selective pressure [66]. Spatial factors also play a role, as ARG abundance was found to be 5–50 times higher near WWTP discharge points than at upstream or distant sites in both the Wenyu and Qinghe Rivers in China [64]. Moreover, resistance to multiple antibiotic classes—including trimethoprim, quinolones, β-lactams, sulfamethoxazole, tetracyclines, and sulfonamides—has been consistently observed in sewage sludge and effluent [68,69,70] (Table S1).

Although WWTPs can substantially reduce the absolute abundance of ARB and ARGs through sedimentation, biodegradation, and disinfection, they can also enrich specific resistant strains or genes, making their role dual and somewhat paradoxical. Selective pressures, microbial competition, horizontal gene transfer (HGT), and biofilm formation in bioreactors collectively contribute to maintaining ARG pools in effluents [71,72].

Recent technological advances aim to mitigate this problem. Advanced oxidation processes (AOPs), photocatalytic degradation, constructed wetlands coupled with UV/O_3_ treatment, and microalgae-based systems have shown promising ARG removal efficiencies under laboratory conditions [73,74,75]. However, large-scale implementation remains limited by high operational costs and energy requirements.

Future research should focus on (i) elucidating the mechanisms governing ARG persistence and horizontal transfer in full-scale WWTPs, (ii) optimizing operational parameters for resistance mitigation, and (iii) developing cost-effective, sustainable technologies that are suitable for practical applications. Integrating molecular surveillance, metagenomics, and resistome risk modeling could provide deeper insights into ARG dissemination and guide next-generation wastewater management strategies.

### 2.2. Fate of ARB and ARGs in Activated Sludge

Activated sludge in wastewater treatment plants is a significant reservoir for ARGs and ARB. Because sludge or sludge compost is frequently applied to agricultural lands, it is critical to measure the abundance and antibiotic tolerance of ARB/ARGs in activated sludge, as well as to understand how physical, chemical, and operational variables affect their fate. Recent work shows that environmental stressors such as nanoplastics can markedly increase ARG abundance in activated sludge. For example, exposure to polystyrene nanoplastics (0.5–50 mg/L) increased relative ARG levels by ~46–59% and enhanced the enrichment of host bacteria (particularly Proteobacteria), mobile genetic elements (MGEs), and genes associated with HGT [64]. Innovations in sludge treatment have demonstrated promising removal of ARGs. Medium-chain fatty acid (MCFA) production processes reduced ARG levels by ~81.2%, limiting ARG host populations and decreasing both vertical and horizontal gene transfer [76]. Similarly, thermal hydrolysis pretreatment (THP) combined with mesophilic anaerobic digestion heavily reduced ARGs (by up to ~3 log units), especially when solids’ retention times and pretreatment temperatures were optimized [77]. However, legacy and earlier studies remain important. For example, Zhang et al. (2018) showed that the soil type exerts a stronger influence on the persistence of antibiotic resistance from composted sludge than compost type [78]. Reinthaler et al. (2003) and Ge, Guo et al. (2012) reported high levels of multidrug resistance in sludge communities. Tetracycline resistance, in particular, has been widely documented in activated sludge across WWTPs [79,80].

In sum, activated sludge processes can reduce the absolute abundance of many ARGs, but they often do not eliminate them, and some ARGs or resistant bacterial populations can persist or even amplify under certain conditions. Key factors influencing fate include pretreatment type and intensity, retention times, sludge composition, exposure to co-stressors (e.g., nanoplastics or heavy metals), and the prevalence of mobile genetic elements. To improve environmental safety, future research should prioritize full-scale trials of promising pretreatment technologies, a better understanding of ARG rebound phenomena, and quantification of the risks posed by exDNA and persistent ARG hosts in sludge applied to land.

### 2.3. Fate of ARB and ARGs in Agricultural Environment

A considerable body of research has documented the presence of ARB and ARGs in RWW from WWTPs worldwide [81,82,83]. The routine use of RWW for irrigation can act as a conduit for the dissemination of ARB and ARGs in agroecosystems [33,84,85,86]. This process poses a potential threat to human health, as human-associated pathogens or opportunistic bacteria may acquire resistance genes and become more difficult to treat [87]. Accordingly, the implications for food safety and public health of applying RWW contaminated with ARB/ARGs to farmland have attracted increasing interest in the scientific community [88,89,90,91].

Fehrenfeld et al. examined ARG distribution at a reclaimed water application site in the western U.S. They found that RWW flowing through the irrigation network still carried a diversity of ARGs, indicating that ARB may regrow downstream of WWTPs. They also detected *gadAB* and *Lmip* genes (markers for *E. coli* and *Legionella pneumophila*, respectively) in the applied RWW, and reported *sul1* and *sul2* among ARGs in soil regimes receiving sequential irrigation with secondary-stage treated wastewater [92].

However, the degree to which ARB and ARGs persist, propagate, or dissipate in soils irrigated with RWW remains contentious and appears to vary by context. Gatica & Cytryn (2013) reviewed multiple field studies and concluded that, in many cases, RWW irrigation does not significantly elevate antibiotic resistance levels in the soil microbiome [85]. In support, Negreanu et al. (2012) studied two soil types in Israel that were irrigated for 6–18 years, either with secondary effluent or with freshwater [93]. They found that the ARB/ARG levels in both soils were lower than what previous literature had suggested and, in most cases, that soils irrigated with RWW had similar or even lower ARB/ARG burdens than those irrigated with fresh water. They argued that the introduced ARB from RWW may poorly survive in agricultural soils and contribute negligibly to ARG transfer.

In contrast, several studies have observed increased ARG abundance and diversity in urban and agricultural soils exposed to RWW. For example, in Beijing, urban parks irrigated with RWW showed elevated abundances of *tetW*, *tetG*, *sul1*, and *sul2*, with the *intl1* integrase gene correlating positively with the *sul* and *tet* genes. Bacteria carrying *sul2* and *intl1* were associated with potential human pathogens (e.g., *Klebsiella oxytoca*, *Acinetobacter baumannii*, and *Shigella flexneri*) [42,94,95,96].

Plants exposed to antibiotics may exhibit physiological and developmental stress responses, including reduced germination, lower chlorophyll content, declined photosynthesis, oxidative stress, tissue deformation, cellular damage, or even mortality. To mitigate these effects, plants deploy detoxification and defense mechanisms such as the induction of cytochrome P450 enzymes and glutathione-S-transferases (GSTs) at the transcriptional and enzymatic levels. They also engage metabolic pathways (hydrolysis, oxidation, reduction, and conjugation) to detoxify or extrude antibiotic molecules via vacuolar sequestration or programmed cell death [97]. In the model plant *Arabidopsis thaliana*, exposure to chlorotetracycline disrupted calcium homeostasis and produced severe stress symptoms in both shoots and roots [97,98]. However, most plant toxicity studies are conducted under laboratory conditions by using spiked antibiotic concentrations [99,100], with relatively few greenhouse or field trials employing realistic mixtures of slurries, manures, or authentic environmental matrices [101,102]. The actual effects in field environments depend heavily on soil pH, antibiotic biodegradation rates, soil organic matter content, chemical speciation, compound bioavailability, and interactions with other soil constituents or pollutants.

## 3. Detection and Quantification of ARB, ARGs, and Antibiotics

**Figure 2:**
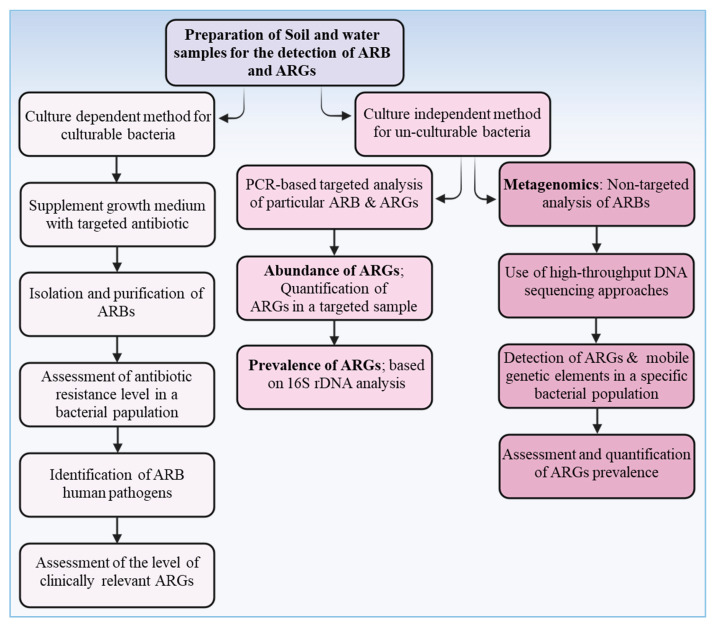
Techniques used for the detection and quantification of ARB and ARGs in the antibiotic-contaminated environment. Created in BioRender. Khalid, A. (2026) https://BioRender.com/anp7zv3.

Although it is technically possible to identify and quantify ARB, ARGs, and antibiotics in soils and crop plants, doing so is often challenging and laborious. Advances in analytical techniques and instrumentation, however, have greatly improved feasibility and precision. Historically, two broad classes of methods have been employed: (1) cultivation-based (phenotypic) methods, which isolate and grow bacteria on selective media to assess antibiotic resistance, and, (2) molecular (culture-independent) methods, which analyze DNA or RNA directly from environmental samples to detect and quantify ARGs and related genetic elements [33,34,69,103] (Figure 2). In this section, we describe the extraction protocols and chromatographic techniques for antibiotics and then focus in Section 3.1 on the detection and quantification of ARB and ARGs in soils, water, and crops.

### 3.1. Detection and Quantification of ARB and ARGs in the Soil, Water, and Crop Plants

The survival and abundance of ARB and ARGs in RWW, soil, and plant tissues can be assessed either via culture-based techniques or by targeting total environmental DNA/RNA (Figure 2). Conventional culture-based methods have limitations (they are labor-intensive, time-consuming, and biased); however, they still offer unique strengths, such as (1) they provide a phenotypic confirmation of resistance (i.e., the bacteria actually survive antibiotic exposure, not merely harbor a gene), and (2) they allow for an assessment of cell viability, isolate characterization, and direct linking of resistance genes to specific bacterial hosts. More modern, efficient approaches rely on molecular techniques that detect ARGs regardless of cultivability. The most widely used are quantitative PCR (qPCR) and metagenomics [37,104,105]. qPCR is highly sensitive (detecting even low copy numbers), and, if well calibrated, can provide absolute quantification of a target gene in an environmental sample. Usually, ARG abundance is normalized to the number of 16S rRNA gene copies (i.e., the ratio of ARG to the total bacterial load). Metagenomics, particularly shotgun sequencing, permits the broad-spectrum detection of ARGs, mobile genetic elements (MGEs), and microbial taxa in a single experiment, thereby capturing the resistome and its ecological context [45]. However, its sensitivity for low-abundance ARGs can be lower than that of qPCR [106]. Hybrid approaches or improvements are emerging (e.g., high-throughput qPCR arrays, droplet digital PCR, microfluidic qPCR) that aim to combine sensitivity with broad coverage [107].

In comparative analyses, qPCR consistently demonstrates higher sensitivity and lower detection limits for targeted ARGs, making it particularly suitable for quantifying low-abundance or clinically relevant resistance markers in complex environmental matrices such as wastewater and soils [105]. Its high throughput, reproducibility, and relatively low cost further support its application in routine monitoring and large-scale surveillance programs. However, qPCR is inherently limited by its reliance on predefined primers, restricting detection to known ARGs and providing little to no information on genetic context, host identity, or novel resistance determinants. In contrast, metagenomic approaches offer a comprehensive, non-targeted view of the environmental resistome, enabling the identification of both known and emerging ARGs, as well as insights into their genomic context, co-localization with mobile genetic elements, and potential host organisms [108,109]. This capability is critical for understanding horizontal gene transfer dynamics and the ecological drivers of resistance dissemination. Nonetheless, metagenomics typically exhibits lower sensitivity for rare genes due to sequencing depth limitations, higher costs, and more complex bioinformatics requirements, which may constrain its routine application [109].

Given these complementary strengths and limitations, a tiered or integrative approach is increasingly advocated in antimicrobial resistance (AMR) surveillance. In such frameworks, qPCR is employed for rapid, sensitive quantification of priority ARGs, while metagenomics is used selectively to provide deeper insights into resistome structure, diversity, and evolutionary potential [109]. The integration of both methods not only enhances detection accuracy but also improves risk assessment by linking ARG abundance with ecological context and potential for transmission. This combined strategy is particularly valuable in environmental systems such as WWTPs and reclaimed wastewater reuse scenarios, where both quantitative precision and mechanistic understanding are essential for evaluating the fate and risks of antibiotic resistance.

Previously, knowledge about ARB and ARGs inside plant tissues (endosphere) was limited. In recent years, however, multiple studies have reported the uptake of antibiotics by plants and the occurrence of ARB/ARGs endophytically [110,111,112,113].

### 3.2. Antibiotics in Soil and Plants

The detection and quantification of antibiotics in environmental matrices, particularly in agricultural soils and plants, are essential for understanding their dissemination pathways and potential impacts on ecosystems, animals, and human health. Our ability to detect macro- and micropollutants in complex environments has advanced significantly due to innovations in analytical chemistry and instrumentation. However, despite this progress, a lack of standardized and validated analytical protocols still limits data comparability and reliability across studies [114,115].

A major analytical challenge lies in the low concentrations of antibiotics in soils and plant tissues and the complex composition of these matrices, which often contain pigments, waxes, lipids, organic matter, and fatty acids that interfere with extraction and detection. An accurate assessment of antibiotic uptake, translocation, and distribution in plants requires a comprehensive analysis of multiple plant organs, including roots, stems, leaves, flowers, and fruits. Thus, the development of matrix-dependent, validated extraction and quantification methods remains a critical need in environmental risk assessment.

#### 3.2.1. Factors Affecting Antibiotic Extraction and Recovery

The physicochemical properties of antibiotics, such as polarity, solubility, and ionization, strongly influence their extractability and quantification efficiency. Moreover, interactions with soil components, including clay minerals and organic matter, can severely reduce recovery. For instance, tetracyclines form strong chelation complexes with metal ions and humic substances, leading to poor extraction and reproducibility [116]. Similarly, the pH of the extraction medium significantly affects the recovery of fluoroquinolones and tetracyclines from both soil and vegetable samples [117].

Another key consideration is the transformation of antibiotics into metabolites or degradation products, known as transformation products (TPs). Many TPs retain biological activity or exhibit enhanced mobility and persistence compared to their parent compounds [118,119]. Unfortunately, analytical detection of TPs remains limited, and only a few multi-residue methods have been optimized for their identification in environmental samples. The development of comprehensive, high-sensitivity analytical platforms, such as high-resolution mass spectrometry (HRMS) and liquid chromatography–tandem mass spectrometry (LC-MS/MS), is thus vital for identifying both parent antibiotics and their transformation products in complex environmental matrices [120].

#### 3.2.2. Sampling and Extraction Techniques

Reliable quantification of antibiotics depends heavily on proper sampling design. Site selection, sampling depth, and methodology (e.g., stratified or composite sampling) must ensure representativeness. Sample preprocessing steps such as cutting, blending, grinding, sieving, and lyophilization are crucial for homogenization and reproducibility. A variety of extraction techniques have been developed for environmental and plant matrices, each with distinct advantages (Table S2). Commonly employed methods include (1) Ultrasound-Assisted Solvent Extraction (USE); (2) Solid-Supported Liquid Extraction (SLE); (3) Pressurized Liquid Extraction (PLE); (4) Quick, Easy, Cheap, Effective, Rugged, and Safe (QuEChERS).

These methods have been effectively applied in the extraction of antibiotics from soils, reclaimed wastewater, and crop plants [121,122,123,124]. The choice of solvent system is also critical. Combinations of water, methanol, ethyl acetate, acetonitrile, and acetone, often supplemented with chelating agents (e.g., EDTA) or buffer systems (e.g., citrate/McIlvaine buffer), are used to prevent metal–antibiotic chelation and improve recovery of compounds such as tetracyclines [125].

#### 3.2.3. Advances in Extraction Protocols

In recent years, the QuEChERS method, originally developed for pesticide extraction, has been widely adapted for the recovery of antibiotics and other emerging contaminants from agricultural samples [126,127]. Modified QuEChERS protocols have significantly improved recovery rates and reduced matrix interference. For example, Hu et al. (2014) optimized the QuEChERS approach for the detection of 26 veterinary antibiotics in vegetables by using an acetonitrile–methanol (85:15 *v/v*) solvent system, achieving up to a 60% increase in recovery for 23 of the target compounds, including tetracyclines and fluoroquinolones [128]. More recent refinements involve the integration of dispersive solid-phase extraction (d-SPE) cleanup steps and coupling with LC–MS/MS or HRMS detection, enabling multi-residue screening of over 50 antibiotics and TPs in a single analytical run [129,130].

Moving forward, the analytical community faces the following three major challenges: (1) standardization of methods across laboratories and matrices to ensure comparability and reliability; (2) expansion of analytical scope to include metabolites, conjugates, and transformation products; and (3) integration of advanced data analytics and machine learning tools for compound identification and source tracking.

Efforts toward global inter-laboratory validation are expected to improve reproducibility. In parallel, the use of isotope-labeled internal standards and automated sample preparation systems will further accelerate progress in understanding the fate of antibiotics in agricultural ecosystems.

## 4. Risk Assessment of ARB, ARGs, and Antibiotics

The environmental occurrence of ARB, ARGs, and residual antibiotics poses a serious threat to the ecosystem and human health. Assessing their potential risks requires a systematic approach that involes hazard identification, exposure analysis, and risk characterization. Although considerable progress has been made, risk assessment frameworks for ARB and ARGs remain underdeveloped due to the biological complexity of microbial systems and the lack of quantitative data on exposure pathways.

### 4.1. Steps for the Risk Assessment

Risk assessment typically involves the following three key stages: (1) hazard identification and characterization, recognizing ARB, ARGs, and antibiotic residues as potential threats; (2) exposure assessment, quantifying the level and frequency of human and environmental exposure to these agents; and (3) risk characterization, integrating hazard and exposure data to evaluate the likelihood and severity of adverse effects (Table S3). In the context of public health, a risk assessment for antibiotic resistance is particularly challenging because it involves living vectors (bacteria) and their mobile genetic elements (MGEs), which can evolve, transfer, and persist across environmental compartments. Despite efforts to develop standardized protocols, guidelines for assessing the dissemination of ARB from environmental reservoirs to humans are still in their infancy [131,132]. Moreover, risk quantification remains uncertain because many influencing parameters, such as gene mobility rates, horizontal transfer efficiency, and survival kinetics in various matrices, are not yet fully measurable or predictable. Table S3 summarizes the critical factors involved in assessing the risk of ARB and ARG transmission from environmental sources to human populations.

### 4.2. Characterization of Environmental Compartment

The first step in the risk assessment process is the detection and quantification of ARB and ARGs within specific environmental compartments, such as soil, water, and WWTPs. Techniques like qPCR, metagenomics, and shotgun sequencing have been extensively used for this purpose [133,134]. An environmental compartment is considered potentially hazardous to human health when vector bacteria are present in sufficient abundance to enable colonization or infection of the human host. The threshold level of concern depends on the type of vector organism, its pathogenic potential, and its resistance profile. For instance, detection of clinically relevant species such as *Klebsiella pneumoniae*, *Enterococcus faecium*, or *Pseudomonas aeruginosa* carrying transferable ARGs in agricultural soils or reclaimed wastewater suggests a higher level of risk [135]

Furthermore, environmental parameters—including pH, temperature, nutrient load, and organic matter—can significantly influence the survival and persistence of ARB and ARGs. For example, ARGs can remain functional for extended periods in sediment or sludge matrices, even in the absence of selective antibiotic pressure [136]. This persistence complicates risk modeling and necessitates the inclusion of environmental variables in exposure assessment frameworks.

### 4.3. Characterization of Vector Bacteria

An accurate risk assessment also requires species- or strain-level identification of vector bacteria. This information allows for the prediction of key biological characteristics such as colonization ability, pathogenic potential, and genetic features, including existing ARG pools and their potential for horizontal gene transfer (HGT). However, this characterization remains incomplete without information on the infective dose (the minimum number of bacterial cells required to cause infection in a host).

**Figure 3:**
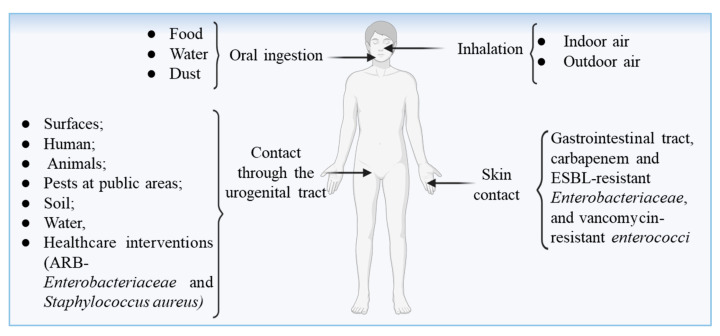
The possible entry and transmission routes of ARB and ARGs from the environment into the human body. Created in BioRender. Khalid, A. (2026) https://BioRender.com/anp7zv3.

Available evidence suggests that even very low concentrations of some ARB can successfully colonize and infect humans. Unfortunately, the infective dose and transmission routes for most clinically relevant ARB, such as *Klebsiella pneumoniae*, *Enterococcus faecium*, *Pseudomonas aeruginosa*, and *Enterococcus faecalis*, remain poorly defined [137,138]. Moreover, antibiotic-resistant clones of these species may exhibit altered infective doses due to changes in virulence and persistence mechanisms. According to the silent colonization model, sub-infective doses of ARB can still colonize the human gut or skin (Figure 3) without causing immediate symptoms, yet they pose a long-term risk through the horizontal transfer of resistance genes to commensal or pathogenic microbiota [85]. Thus, even minimal exposure can have far-reaching implications for resistance dissemination.

ARB, with a low infective dose, high-resistance gene content, and strong replication potential, are considered the highest risk category. Unfortunately, current molecular methods, such as qPCR and metagenomics, while powerful, may not detect low-abundance or non-culturable resistant populations, leading to an underestimation of the actual risks [139].

### 4.4. Emerging Evidence and Public Health Implications

Recent studies have highlighted an alarming increase in antibiotic resistance among environmental and clinical isolates of *Pseudomonas aeruginosa* (CRPA-VIM), *Escherichia coli*, and *Acinetobacter baumannii*, emphasizing the interconnection between environmental reservoirs and human infections [140,141]. ARB originating from environmental sources such as wastewater, sludge, or agricultural runoff have been directly linked to clinical infections, hospital outbreaks, and even fatalities [142,143].

The coexistence of ARB and ARGs in environmental matrices exerts a profound impact on microbial community structure and function, promoting horizontal gene transfer and the evolution of novel resistance determinants [144]. Moreover, the release of human-associated microbiota through untreated or partially treated wastewater amplifies the opportunity for cross-domain gene exchange, accelerating the environmental spread of resistance [85,145]. To effectively assess these risks, integrating conceptual and quantitative models is essential. Conceptual frameworks identify the sources, pathways, and sinks of antibiotics, ARB, and ARGs across interconnected compartments such as WWTPs, RWW, soils, and receiving waters. Building on this, quantitative approaches like quantitative microbial risk assessment (QMRA) estimate human health risks by linking exposure routes (e.g., irrigation, water reuse, and food consumption) to dose–response relationships. QMRA can incorporate ARB and ARG abundance, horizontal gene transfer dynamics, and treatment performance to generate probabilistic risk estimates [146]. Recent advances integrating fate and transport models with genomic data further improve predictions of resistance dissemination and persistence [131]. However, uncertainties remain, particularly regarding dose–response relationships for ARGs and complex microbial interactions. Thus, combining conceptual insights with robust quantitative tools is crucial for developing risk-based management strategies and guiding policies to mitigate the environmental spread of antibiotic resistance.

Developing a comprehensive risk assessment framework for ARB and ARGs requires the following: (1) establishing standardized protocols for detection, quantification, and data reporting; (2) incorporating metagenomic and bioinformatics tools for detailed microbial profiling; (3) defining infective dose thresholds for major ARB species; and (4) integrating environmental and epidemiological data into global surveillance systems.

Such efforts, coupled with international collaboration and data harmonization, will be crucial for quantifying the true impact of environmental antibiotic resistance and for informing effective policies on wastewater reuse, sludge management, and agricultural practices.

### 4.5. Indicators and Metrics for Risk Quantification

Robust indicators and metrics are critical for quantifying the environmental and public health risks associated with antibiotics, ARB, and ARGs. Among these, disability-adjusted life years (DALYs) provide a comprehensive measure of disease burden by integrating both morbidity and mortality, enabling a comparison of antibiotic resistance impacts with other environmental health risks [147]. In parallel, probabilistic metrics derived from QMRA, such as the probability of infection or illness, are widely used to estimate human exposure risks under different scenarios, including wastewater reuse and agricultural irrigation [146,148]. Additional indicators, such as the abundance and diversity of ARGs, the frequency of HGT, and resistome profiling indices, are increasingly employed to assess environmental dissemination and persistence [131]. Integrating these metrics within risk assessment frameworks allows for a more holistic evaluation of both direct health outcomes and indirect ecological impacts. However, challenges remain in harmonizing indicators, particularly in linking ARG occurrence to clinical outcomes and establishing standardized thresholds. Therefore, the development of unified, multi-metric approaches is essential for improving risk characterization and supporting evidence-based decision-making in the management of antibiotic resistance in the environment.

Building on these quantitative frameworks, emerging techniques such as metatranscriptomics and functional resistomics further enhance risk assessment by providing functional and mechanistic insights. While DNA-based methods (e.g., qPCR and metagenomics) characterize ARG presence and diversity, metatranscriptomics reveals actively expressed genes, enabling a more accurate evaluation of real-time risks in environments such as WWTPs and RWW [149]. Complementarily, functional resistomics—based on heterologous expression and screening—uncovers novel ARGs and links genotype to phenotype, overcoming key limitations of sequence-based approaches [150]. Despite challenges related to cost and data complexity, integrating these advanced techniques with established indicators and models provides a more comprehensive and functionally relevant understanding of the environmental resistome, ultimately strengthening risk assessment and mitigation strategies.

## 5. Risk Factors and Mode of Transmission

The transmission pathways of antibiotic resistance from environmental sources to humans remain insufficiently understood. The complexity of microbial interactions, coupled with anthropogenic pressures, contributes to the spread and persistence of antibiotic resistance elements (AREs) in natural and engineered ecosystems.

### 5.1. The Antibiotic Resistome: Natural Versus Contaminated

**Figure 4:**
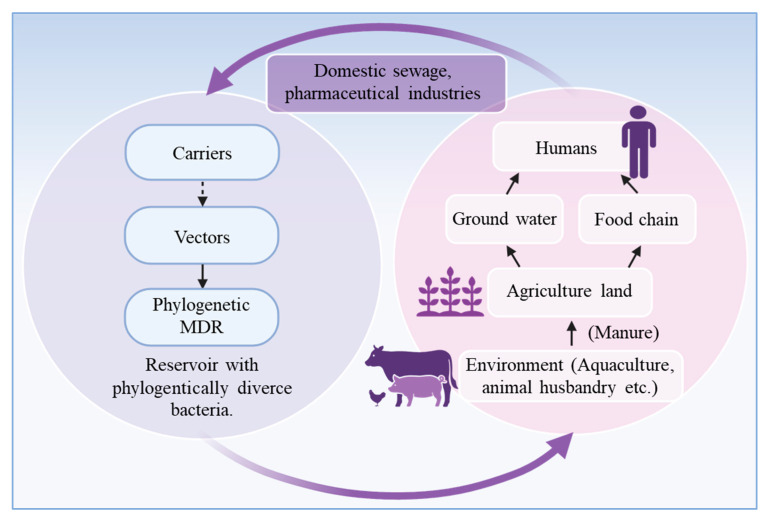
Schematic diagram of the relationship between natural and contaminated environmental resistome, responsible for the translocation of ARB and ARGs from the environment to humans and back to the reservoir. Created in BioRender. Khalid, A. (2026) https://BioRender.com/anp7zv3.

The environmental resistome encompasses both the natural reservoir of antibiotic resistance genes (ARGs) and the resistome that has evolved or expanded due to anthropogenic activities (Figure 4). In the natural resistome, many antibiotic-resistant bacteria (ARBs) share genetic similarity with clinically significant multidrug-resistant (MDR) pathogens, suggesting that environmental bacteria may serve as the evolutionary origin of antibiotic resistance [151] These environmental ARBs act as reservoirs of resistance genes [152], hosting phylogenetically diverse bacterial taxa—often from antibiotic-producing or resistant phyla such as *Proteobacteria*, *Actinobacteria*, and *Bacteroidetes* [153,154].

**Figure 5:**
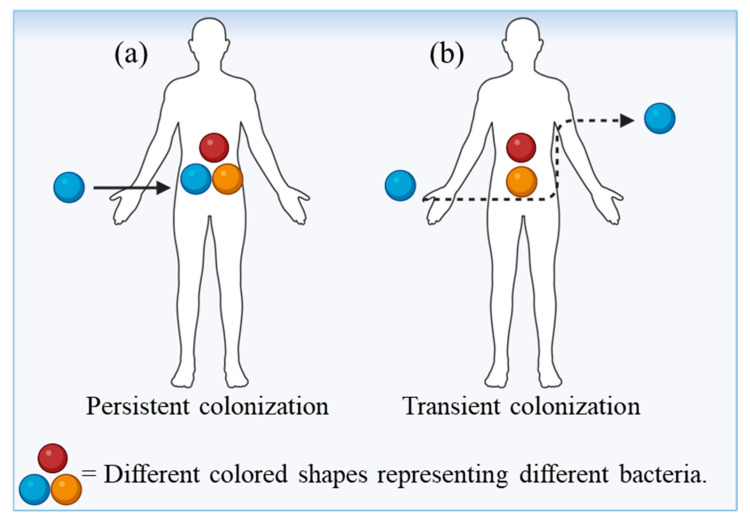
Colonization of antibiotic-resistant bacteria (the vectors): (**a**) persistent colonization; (**b**) transient colonization. Created in BioRender. Khalid, A. (2026) https://BioRender.com/anp7zv3.

The resistome typically consists of two major functional groups: (i) carriers—ARBs that disseminate ARGs in the environment but cannot infect humans directly; and (ii) vectors—ARBs capable of invading and colonizing the human body (Figure 5).

These groups are not necessarily distinct taxonomically but differ in ecological behavior and physiological adaptations. Members of the classes *Betaproteobacteria* and *Gammaproteobacteria*, as well as the phyla *Firmicutes* and *Actinobacteria*, have been frequently identified as both vectors and carriers [154,155]. Genera such as *Aeromonas*, *Acinetobacter*, *Pseudomonas*, and *Staphylococcus*, and members of the *Enterobacteriaceae* family serve as important reservoirs; some species also function as vectors [155,156] (Table S1). Although environmental carriers do not colonize the human body, their proliferation in water, soil, and agricultural systems increases the abundance of ARGs in potential vector populations, thereby enhancing the risk of HGT and the eventual transmission of antibiotic resistance to humans [157,158].

### 5.2. Vector Bacteria (A Source of ARG Transmission from the Environment to Humans)

Vector bacteria are capable of transiently or permanently colonizing humans and can play a crucial role in the environmental transmission of ARGs (Figure 4 and Figure 5). To successfully colonize, these bacteria must survive and persist in environments where they frequently encounter humans, such as wastewater, agricultural soils, or food systems. Evidence indicates that multiple environmental bacterial lineages overlap with those found in the human microbiome, emphasizing the permeability of ecological and clinical boundaries [155]. The impact of vectors on human health depends on several factors, including the host’s immune status, microbial competition, and the vector’s capacity to proliferate within host tissues. Vectors harboring multiple ARGs and virulence determinants are often termed “superbugs,” representing a severe public health threat due to their resistance to multiple antibiotic classes (Figure 4). These MDR pathogens can transfer ARGs through plasmids, bacteriophages, free DNA uptake, and other horizontal gene transfer mechanisms such as transformation, conjugation, and transduction, enhancing their genetic diversity and adaptability. Notably, several vector species with broad-spectrum resistance, such as extended-spectrum β-lactamase (ESBL)-producing Enterobacteriaceae, multidrug-resistant *Clostridium difficile*, *Pseudomonas aeruginosa*, methicillin-resistant *Staphylococcus aureus* (MRSA), and vancomycin-resistant *Enterococci* (VRE) are prioritized by global antimicrobial resistance surveillance programs [159].

## 6. Concluding Remarks and Future Directions

Wastewater treatment plants (WWTPs) play a critical role in reducing the abundance of antibiotics, antibiotic-resistant bacteria (ARB), and antibiotic resistance genes (ARGs). However, despite these efforts, WWTPs often remain hotspots for the persistence, dissemination, and environmental transmission of resistance elements, highlighting the urgent need for eco-friendly, cost-effective, and broadly applicable strategies to enhance treatment efficiency and limit their environmental spread.

Equally important is addressing the dispersal of antibiotic resistance in agricultural and livestock systems to ensure the production of safe and healthy food. On-site field studies are particularly needed to evaluate the prevalence, persistence, and proliferation of ARBs and ARGs in soils and irrigation systems. Current knowledge of phytotoxicity and plant stress responses induced by antibiotics, ARBs, and ARGs is largely based on laboratory or greenhouse experiments, which may not accurately reflect complex field conditions. This limitation underscores the need for comprehensive, field-based investigations that capture real-world environmental dynamics and provide more reliable assessments of agronomic and ecological risks.

A major challenge in synthesizing current evidence is the lack of methodological standardization across studies. Variations in sampling strategies, detection techniques (e.g., qPCR, metagenomics, culture-based methods), and reporting metrics limit comparability and hinder the development of generalized conclusions. Furthermore, uncertainty in quantitative risk assessment remains high, particularly in linking ARG abundance to actual human health outcomes. Existing techniques, although valuable, are often insufficient for fully reliable estimation of exposure risks from environmental reservoirs to humans.

To address these gaps, future research should adopt integrative, interdisciplinary approaches that combine high-resolution metagenomics, culture-dependent analyses, metatranscriptomics, functional resistomics, immunological assays, and advanced bioinformatics. These combined approaches can improve our understanding of the ecology, mobility, and transmission potential of ARB and ARGs within complex environmental compartments. Incorporating standardized metrics and risk indicators, such as ARG abundance, HGT frequency, and probabilistic exposure models, will further enhance risk characterization and reduce uncertainty.

Ultimately, bridging these knowledge gaps will enable the development of holistic management strategies that are aimed at reducing the environmental burden of antibiotic resistance, safeguarding public health, and supporting sustainable agricultural and wastewater practices. Emerging technologies, including resistome-wide surveillance, advanced wastewater treatment innovations, and functional monitoring of ARG activity, are poised to play a transformative role in achieving these goals, provided they are implemented within standardized and rigorous methodological frameworks.

## Data Availability

No new data were created or analyzed in this study. Data sharing is not applicable to this article.

## Supplementary Materials

The following supporting information can be downloaded at https://www.mdpi.com/article/10.3390/ijms27073255/s1, References [160,161,162,163,164] are cited in the Supplementary Materials.
