# Supplementary material for: Environmental Risks of Antibiotics and Antibiotic Resistance Elements: Occurrence, Fate, and Assessment
Source: Int J Mol Sci. 2026 Apr 3;27(7):3255. doi: 10.3390/ijms27073255 (PMC13073191; doi:10.3390/ijms27073255)
Supplement: Supplementary file 1 [file ijms-27-03255-s001.zip › ijms-4217257-supplementary.pdf]

Environmental Risks of Antibiotics and Antibiotic Resistance Elements: Occurrence, Fate, and Assessment

Fiaz Ahmad [1,*], Azzam Fatima Zahra [1], Noreen Ashraf [1] and Zafar Iqbal [2]

[1] Key Laboratory for Space Bioscience & Biotechnology, School of Life Science and Technology, Northwestern Polytechnical University, Xi'an 710129, China
[2] Central Laboratories, King Faisal University, Al-Ahsa 31982, Saudi Arabia

* Correspondence: fiaz.a@mail.npu.edu.cn

Abstract

Antibiotics, antibiotic-resistant bacteria (ARB), and antibiotic resistance genes (ARGs) have emerged as critical environmental contaminants posing serious ecological and public health concerns. The widespread occurrence and proliferation of ARB and ARGs in wastewater treatment plants (WWTPs) and reclaimed wastewater (RWW) used for irrigation represent major pathways for their dissemination into the environment. Current knowledge indicates that ARGs from the environmental resistome can be transferred among diverse microbial communities, including clinically relevant human pathogens. Numerous studies have also linked the expansion of the environmental resistome to anthropogenic activities. Therefore, preventing and mitigating the spread of antibiotic resistance in the environment requires a deeper understanding of how resistance genes evolve, transfer, and persist across ecological compartments. This review synthesizes the current state of knowledge on the occurrence, prevalence, and detection of antibiotics, ARB, and ARGs in various environmental matrices, providing essential insights for developing preventive strategies and promoting the sustainable management of ecosystems.

Keywords: antibiotic resistance; environmental resistome; risk assessment; public health; dissemination

Table S1. Antibiotic-resistance of the microbial community in WWTPs.

Antibiotic-resistant Bacteria (ARB)	Effect	Sampling Source	References
Sulfonamide and Tetracycline-resistant bacteria	Elevated resistance found in treated WWRPs effluent	Sewage from five different WWTPs in Michigan	[160]
Tetracycline and sulfonamide-resistant bacteria	The rate of sulfonamide was 2-3 times higher than tetracycline both in surface water and in sediments	7 urban rivers in Beijing, China	[64]
Aeromonas and *Enterobacteriaceae*-resistant bacteria	Showed resistance against 21 out of 22 tested antibiotics	Wastewater of a WWTP in Agra River (Spain)	[59]
Quinolones and b-lactams, tetracycline, quinolones, sulfonamides, trimethoprim and sulfamethoxazole resistant *E. coli*, *Acinetobacter* spp., *P. aeruginosa*, *Pseudomonaades*, and *Enterobacteriaceae*.	WWTPs are the reservoirs for diverse ARB and ARGs	Sewage sludge obtained from WWTPs	[161, 162]
Relative abundance of ARB genera (*Pseudomonas*, *Corynebacterium*, *Ochrobactrum*, *Bacillus*, and *Actinomadura*) and antimicrobial indicator ARGs (*Sul1, sul2, tetB, tetM, ermB, ermF, fexA, cfr, intI1*)	Dramatic changes in ARB and ARGs structure and diversity in response to poultry manure application	Simulated poultry manure-soil	[163]

Table S2. Methods for detection and quantification of antibiotics in plant and soil samples

Methodology/Instrumentation	Analytes	Sample Type	Sample Preparation	% Recovery	LOD/LOQ* ng/g	Reference
Q-Orbitrap LC-MS/MS coupled with Hypersil Gold column (2.1 mm×50 mm, 3 µm)	Hydroxy trimethoprim, trimethoprim, clindamycin, clindamycin sulfoxide, N4-acetyl sulfamethoxazole, sulfamethoxazole, hydroxy clindamycin sulfoxide, s-dimethyl clindamycin, n-dimethyl clindamycin	Soil	Acetonitrile:water (1:1), + 0.1% formic acid	66-123	1.2-7 pmol/g **(MDL) & 3.2-22 pmol/g (MQL)**	[19]
LC-DAD C18 column-Waters with dimensions (30 cm × .32 cm, 0.25 µm)	Chlortetracycline, oxytetracycline, norfloxacin	Soil and rice plant (*Oryza sativa*)	Macilvaine buffer-EDTA extraction & SPE (Strata-X) clean-up	Soil (65-76), & Rice plant (73-78)	Soil - 80-300 MDLs, & Rice plant – 70-450 MDLs	[121]
LC-ESI-QqQ-MS/MS XTerra MS C18 column with dimensions (2.1 mm × 30 mm, 2.5 µm)	Trimethoprim, sulfamethoxazole, ofloxacin	Vegetated and grains of wheat plant (*Triticum aestivum*)	SPE (Oasis HLB) + PLE (MeOH)	44-84	LOD-0.1-0.6<br>LOQ-0.3-1.8	[122]
LC-ESI-QqQ-MS/MS, Zorbox SB-Aq (150 mm × 2.1 mm, 3.5 µm)	**Sulfonamides**; sulfaclozine, sulfaquinoxaline, sulfamonomethoxine, sulfamethoxydiazine,	Vegetables; romaine lettuce, chinese cabbage, white radish	QuEChERS: CAN-MeOH (85:15 v/v) + citric buffer extraction + L-L partition ($MgSo_4$ and NaCl) + dSPE (PSA)	60-98	LOD- 0.005-0.5<br>LOQ- 0.02-1.5	[128]

	sulfamethoxazole, and sulfadimidine. **Macrolides**; tulathromycin, erythromycin, tilmicosin, kitasamycin, tylosin, and roxithromycin. **Tetracyclines**; doxycycline, chlortetracycline, oxytetracycline, and tetracycline. **Fluoroquinolones**; norfloxacin, diflosacin, enoxacin, sarafloxacin, ciprofloxacin, and enrofloxacin. **Lincosamides**; lincosamycin, and clindamycin.	and Fruits; cucumber, string bean, green pepper				
LC-ESI-QqQ-MS/MS Zorbas, Eclipse PLUS C18 column with dimensions (50 mm × 2.1 mm, 1.8 µm)	**Macrolide**; roxithromycin, erythromycin, and tylosin. **Sulfonamides**; sulfadimethoxine, sulphanilamide, sulfadimidine, sulfadiazine, trimethoprim, sulfathiazole, and sulfameter.	Soil	For extraction QuEChERS : AcN + SPE clean-up with polymeric and anion-exchange cartridges	2-114	MLD- 0.007-7<br>MLQ- 0.01-17	[128]

	**b-lactam;** penicillin G, **Anti-parasitic**; dicyclanil and phenicol florfenicol					
HPLC- waters X-Bridge C18 (4.6 mm × 250 mm, 5 µm)	**Sulfonamides**; ^{14}C-ring-labeled Sulfamethoxazole (SMX) and its metabolite ^{14}C-ring-labeled N-acetyl-sulfamethoxazole (N-ac-SMX). **Fluoroquinolone**; ^{14}C-ring-labeled ciprofloxacin,	Soil	Cyclodextrin HPCD + Ultima Gold XR scintillation liquid + AcN:Mc Ilvaine ph9 buffer for extraction. NaCl and HCl were used to adjust the pH.	10-72	-	[164]

LOD = Limit of detection / LOQ = Limit of quantification*, MDL = Method limit of detection & MQL = Method limit of quantification**

Table S3. Steps of risk assessment and critical factors are significantly essential to identify the intensity of risk associated with ARB and ARGs.

Steps of Risk Assessment	Identification of Hazard	Characterization of Hazard	Assessment of Exposure	Assessment of risk
**Description of the steps**	Identification of ARB and ARGs from contaminated resistome originated from anthropogenic activity.	The quantitative and qualitative assessment of ARB and ARGs and their effects on humans.	The quantitative and qualitative assessment of the probability that a vector from the environment can harm somebody.	Based on first three steps, estimation of the adverse effects likely to occur. **4 = 1 × 2 × 3**
**Critical Factors**	**a.** Whether the environment harbors ARB, potentially proliferates and transfer genes horizontally.<br>**b.** Does environment have direct contact with humans? By-using treated wastewater for irrigation purposes on agriculture soil, urban wildlife, and recreational areas.	**a.** Does the ARB proliferate, colonize and invade organs and tissues of humans?<br>**b.** Whether ARB is multidrug-resistant or can resist the high dose of antibiotics	a. Whether the rate of exposure to source identified in (step-1) is high.<br>b. Whether the identified bacteria in step-2 are motile through water, food, air, person-to-person, fomites, and pets.<br>c. Potential of colonization in humans is high.	
